# Cardio–Splenic Relationship in Patients Receiving Trans-Catheter Aortic Valve Replacement

**DOI:** 10.3390/jcm12237392

**Published:** 2023-11-29

**Authors:** Teruhiko Imamura, Hayato Fujioka, Ryuichi Ushijima, Mitsuo Sobajima, Nobuyuki Fukuda, Hiroshi Ueno, Koichiro Kinugawa

**Affiliations:** The Second Department of Internal Medicine, University of Toyama, 2630 Sugitani, Toyama 930-0194, Japanhueno@med.u-toyama.ac.jp (H.U.); kinugawa-tky@umin.ac.jp (K.K.)

**Keywords:** heart failure, hemodynamics, spleen, aortic valve disease

## Abstract

Background: The spleen seems to be a significant buffer of the effective circulating blood volume by changing its size dramatically according to hemodynamics. The cardio–splenic relationship has been recently proposed in the literature regarding heart failure cohorts, and the splenic size has been proposed as a prognostic factor in these cohorts. However, the clinical implication of the splenic size in patients receiving trans-catheter aortic valve replacement (TAVR) remains unknown. Methods: Patients who underwent TAVR at our institute between 2015 and 2022 were eligible. Of these, the patients whose abdominal computed tomography imaging was obtained before TAVR were included. The volume of the spleen was measured in all the participants. The prognostic impact of the indexed splenic volume (SVI) on the 2-year cardiac death or heart failure readmissions was evaluated. Results: A total of 343 patients (86 years, 98 males) were included. The median value of the peak velocity at the aortic valve was 4.4 (4.0, 4.8) m/s and the median SVI was 65.5 (48.9, 86.9) mL/m^2^. A lower SVI tended to be associated with a lower cardiac output, whereas a higher SVI was associated with a higher plasma volume. The SVI was independently associated with the 2-year cardiac death or heart failure readmission after TAVR with a hazard ratio of 1.09 (95% confidence interval 1.01–1.18, *p* = 0.041), adjusted for potential confounders. A calculated cutoff of SVI was 70.2 mL/m^2^, which significantly stratified the cumulative incidence of the primary outcome (10% versus 4%, *p* = 0.033). Conclusion: A high baseline SVI, which was associated with systemic congestion, was associated with a higher incidence of cardiac death or heart failure readmission after TAVR. Further studies are warranted to clarify the detailed cardio–splenic relationship and implication of measuring the SVI in this cohort.

## 1. Background

Trans-catheter aortic valve replacement (TAVR) was introduced as a minimally invasive trans-catheter intervention for the treatment of severe aortic stenosis, primarily targeting patients at elevated surgical valve replacement risk [1]. Subsequent enhancements in clinical outcomes associated with TAVR have been attributed to refined patient selection, dedicated pre-procedural imaging analyses, the advent of next-generation device design, procedural streamlining encompassing single arterial access and conscious sedation, and the shift from dual to single antiplatelet therapy post-TAVR [2,3]. The spectrum of TAVR candidates has broadened to encompass intermediate and lower-risk patients, thereby expanding the existing guideline recommendations for TAVR [4,5,6,7]. Nevertheless, certain patients continue to grapple with elevated mortality and morbidity even following TAVR [8].

One of the pressing unmet needs for individuals deemed suitable for TAVR revolves around the persistence of congestion post-procedure [9]. Post-TAVR residual congestion correlates with recurrent heart failure stemming from volumetric overloading [10]. Ideally, the assessment of the congestion degree before the TAVR procedure should be prioritized for the purpose of engaging in shared decision-making with patients and their families, undertaking risk stratification, contemplating therapeutic strategies, and discussing the TAVR indication in advance.

Several indices are at our disposal to evaluate systemic congestion, encompassing physical examination, estimated plasma volume, and invasively measured right heart catheterization hemodynamic data [11,12,13,14]. Importantly, these parameters are expressed in absolute terms, rendering the threshold for what constitutes “significant congestion” somewhat ambiguous. For instance, a central venous pressure of 12 mmHg might induce noteworthy congestion and resultant end organ damage in one patient but prove innocuous in another. The conundrum of assessing “significant congestion”, which poses a hazard to end organs, has long remained unresolved.

Recently, the cardio–splenic relationship has garnered substantial attention [15]. The spleen, which stockpiles approximately 30% of the total blood volume, serves as a buffer for the systemic fluid volume [16]. In circumstances where the effective circulating blood volume diminishes due to factors such as cardiogenic shock, sympathetic nervous system activation, or end organ ischemia, the spleen endeavors to contract as a means of preserving the systemic volume [17,18,19,20,21]. Conversely, in patients beset by systemic congestion, the spleen endeavors to enlarge itself to accommodate the additional blood influx necessitated by volume overload [22]. Earlier literature has propounded the clinical significance of measuring the splenic size, as it provides insight into the assessment of “significant congestion”.

We posited that an enlarged spleen may signal substantial systemic congestion and could be linked to future occurrences of congestive heart failure following TAVR, which remains an unsolved issue. This study seeks to explore the prognostic impact of baseline splenic size measurements conducted before TAVR on the post-procedural cardiac mortality and the recurrence of heart failure.

## 2. Methods

### 2.1. Patient Selection

Patients with severe aortic stenosis who underwent TAVR at our center between 2015 and 2022 were eligible for this retrospective study. Patients who received abdominal computed tomography imaging on admission according to our institutional protocol were included. Patients without critical data such as echocardiographic and right heart catheterization data were excluded. Patients who died during index hospitalization were excluded given the lack of follow-up data. Patients with lost follow-up data were also excluded.

Written informed consent was obtained from all participants on admission. The institutional review board approved the study protocol (R2015154, 11 April 2016).

### 2.2. Measurement of Splenic Volume

**Figure 1:**
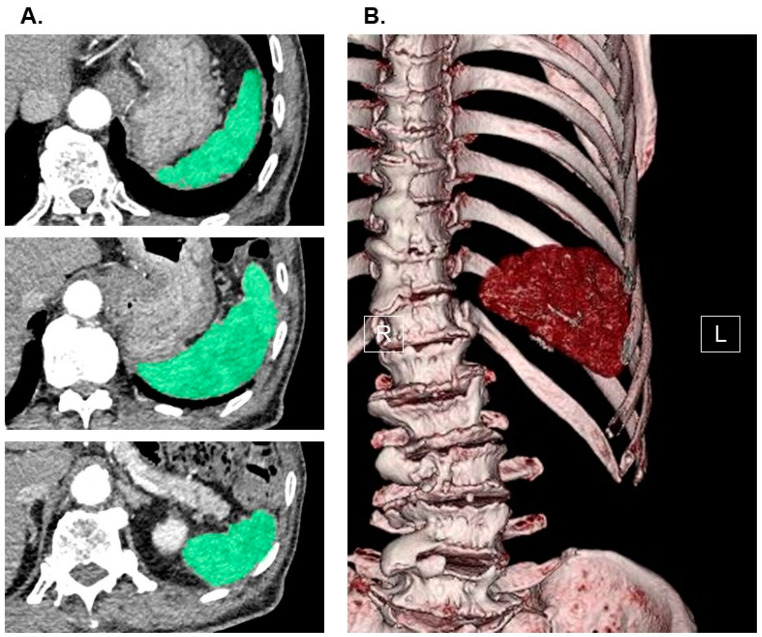
Measurement of splenic volume using abdominal computed tomography imaging. Spleen (green) in horizontal section was traced by the software in each slice (**A**). Three-dimensional image of spleen was displayed (**B**). SYNAPSE VINCENT software, version 2.0003, developed by Fujifilm in Tokyo, Japan, was used to measure splenic volume. Using the software’s preset algorithm for extracting elliptical tissue, the software automatically recognized the spleen when the long axis of the spleen was specified from a multi-slice computed tomography scan cross-sectional image (spleen was traced and colored in green in each slice). All the recognized splenic images were constructed in three dimensions and output as an image, and its splenic volume was automatically calculated. In cases where necessary, manual adjustments were made to the trace before arriving at the final measurements. For the independent variable, we indexed the value of splenic volume. R, right; L, left.

The measurement of splenic volume was carried out through computed tomography imaging, with slices as thin as 1.0 mm, obtained upon admission. This process involved the utilization of SYNAPSE VINCENT software, version 2.0003, developed by Fujifilm in Tokyo, Japan. Using the data from the multi-sliced computed tomography scans, three-dimensional abdominal images were reconstructed. The software employed an algorithm designed to automatically identify the spleen by recognizing elliptical tissue. In cases where necessary, manual adjustments were made to the trace before arriving at the final measurements (for a comprehensive method, please refer to Figure 1). The splenic volume was then normalized for the patients’ body surface area (Du Bois method), resulting in the calculation of the splenic volume index (SVI) as an independent variable.

### 2.3. TAVR Procedure

Patients presenting with severe aortic stenosis characterized by a peak velocity exceeding 4.0 m/s, a mean pressure gradient surpassing 40 mmHg, or an aortic valve area less than 1.0 cm^2^ were deemed eligible candidates for TAVR [23]. The ultimate decision to proceed with TAVR was reached through clinical consensus within a multidisciplinary team, which included cardiac surgeons, interventional cardiologists, anesthesiologists, and imaging specialists. This decision-making process also involved comprehensive informed consent discussions with both the patients and their relatives.

Patients underwent a standard TAVR procedure utilizing the Edwards Sapien XT/Sapien 3 Transcatheter Heart Valve from Edwards Lifesciences, based in Irvine, CA, USA, or the Medtronic CoreValve/Evolut R Revolving System from Medtronic, headquartered in Minneapolis, MN, USA. The TAVR procedure was performed through various routes, including trans-femoral, trans-subclavian, or direct-aorta access, while patients received local or systemic anesthesia as determined by the medical team.

The choice of post-TAVR antithrombotic therapy was left to the discretion of the treating physician. Following a week of meticulous observation and confirmation of the absence of active procedure-related complications, patients were discharged. Subsequent to the index discharge, patients were subject to regular follow-up appointments either at our outpatient clinic or affiliated institutions, supervised by board-certified cardiologists. Standard laboratory and echocardiographic assessments were performed at least once annually.

### 2.4. Independent Variable and the Primary Outcome

The independent variable was defined as SVI obtained before TAVR. The primary outcome under investigation was a composite endpoint comprising cardiac mortality and instances of heart failure readmissions occurring within a 2-year observation period following the index discharge. The occurrence of these events was validated through a meticulous review of medical records or through telephone inquiries.

### 2.5. Other Clinical Variables

Prior to the TAVR procedure, a comprehensive set of baseline characteristics was compiled, encompassing demographic, comorbidity, laboratory, and echocardiographic data. These data were collected as a part of the study’s baseline assessment.

Plasma volume status was assessed employing the Hakim formula, as elucidated earlier, utilizing data related to hematocrit, body weight, and body height (see Appendix A). Day 0 was specifically defined as the moment of index discharge; patients who succumbed during their initial hospitalization were excluded from the analysis due to the absence of follow-up data.

### 2.6. Statistical Analysis

Continuous variables were presented as medians with interquartile ranges (25th and 75th percentiles), while categorical variables were expressed as counts and corresponding percentages. Significance was defined as a two-tailed *p*-value less than 0.05. The statistical analyses were conducted using SPSS Statistics 23, developed by SPSS Inc. in Armonk, IL, USA.

The independent variable in this analysis was defined as the baseline SVI, acquired prior to TAVR procedure. To explore the relationship between SVI and other clinical parameters, patients were divided into tertiles based on their baseline SVI using cutoffs of 54.8 mL/m^2^ and 75.6 mL/m^2^: first, second, and third tertile groups. Comparative analyses of baseline characteristics across these three groups were executed using the Kruskal–Wallis test and post hoc Dunn’s test for continuous variables and the chi-square test for categorical variables.

The primary outcome was defined as the occurrence of either cardiac death or heart failure readmission within the 2-year observation period following the index discharge. The influence of potential baseline characteristics, including SVI, on the primary outcome was assessed through Cox’s proportional hazard ratio regression analysis. Baseline characteristics deemed potential risk factors for the primary outcome were included in this time-to-event analysis. Variables with a *p*-value less than 0.05 in the univariable analysis were incorporated into the multivariable analysis. A receiver operating characteristics analysis was conducted to determine the SVI cutoff value for predicting the primary outcome. Subsequently, patients were stratified into two groups based on this SVI cutoff: a low SVI group and a high SVI group. The cumulative incidence of the primary outcome in these two groups was compared using the log-rank test.

## 3. Results

### 3.1. Baseline Characteristics

A total of 345 patients who underwent TAVR at our institution between 2015 and 2022 met the eligibility criteria. Of these, 343 patients with available abdominal computed tomography imaging to measure the SVI were included (Table 1). The median age was 86 (83, 88) years and 98 were male patients. The baseline median peak velocity at the aortic valve was 4.4 (4.0, 4.8) m/s and the median plasma B-type natriuretic peptide level was 221 (119, 483) pg/mL.

### 3.2. SVI Distribution

**Figure 2:**
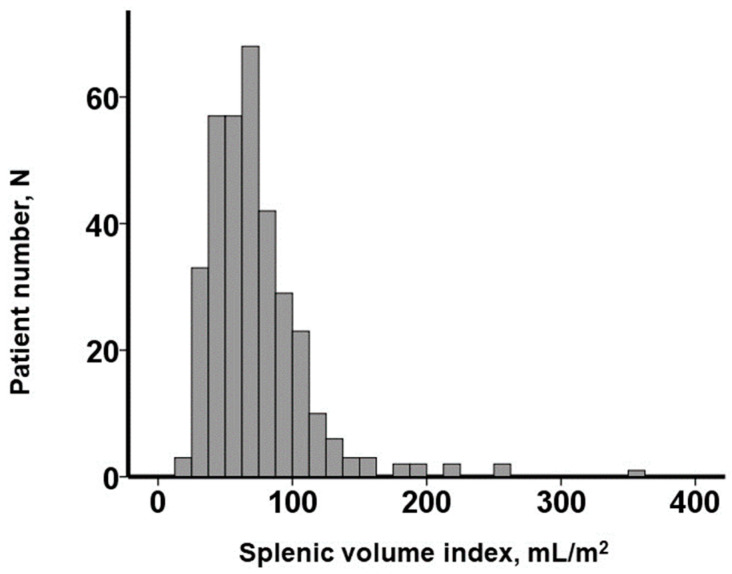
Distribution of splenic volume index obtained before transcatheter aortic valve replacement.

**Figure 3:**
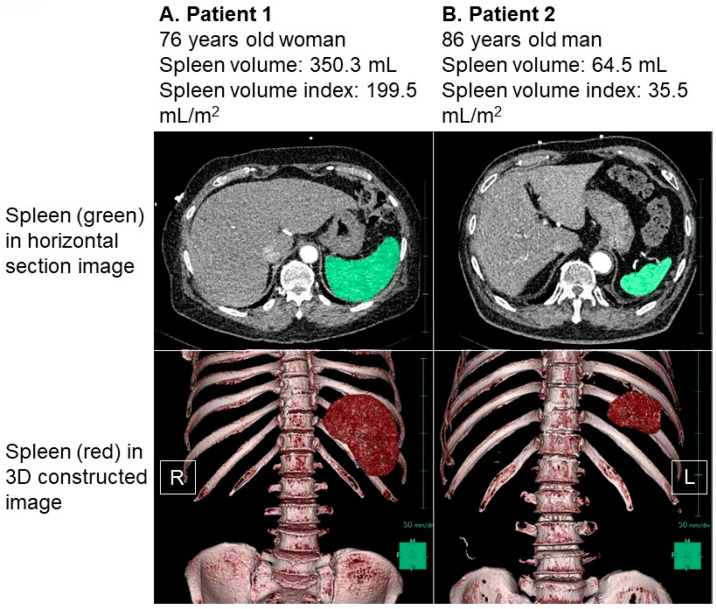
Representative cases of high splenic volume index (**A**) and low splenic volume index (**B**). Spleen was colored in green in horizontal section image and was colored in red in three-dimensional constructed image. (**A**) Patient 1 was 76-year-old woman. Body height was 160 cm and body weight was 72.3 kg. Her splenic volume index was calculated as 199.5 mL/m^2^. (**B**) Patient 2 was 86-year-old man. His body height was 161 cm and body weight was 76.8 kg. His splenic volume index was calculated as 35.5 mL/m^2^. Splenic volume was larger in patient 1 compared to patient 2 in both 2-dimensional and 3-dimenstional imaging. R, right; L, left.

The distribution of the baseline SVI values exhibited considerable variability, with a median SVI of 65.5 (48.9, 86.9) mL/m^2^ (Figure 2). For illustrative purposes, representative cases of a low SVI and a high SVI were presented in Figure 3A and Figure 3B, respectively.

In Figure 3A, we observe a 76-year-old female patient who had a body height of 160 cm and a body weight of 72.3 kg, resulting in a body mass index of 28.2. Her splenic volume was measured at 350.3 mL, and when indexed to her body surface area, it amounted to 199.5 mL/m^2^. Figure 3B showcases an 86-year-old male patient with a body height of 161 cm and a body weight of 76.8 kg. His splenic volume was measured as 64.5 mL, and when indexed to his body surface area, it equated to 35.5 mL/m^2^.

To analyze the patient cohort effectively, we divided it into tertiles based on the SVI cutoffs of 54.8 mL/m^2^ and 75.6 mL/m^2^. A comparison of the patient characteristics across these three groups is detailed in Table 1. It was observed that a lower SVI was associated with a reduced cardiac index (*p* = 0.048). Conversely, a higher SVI was correlated with a higher body mass index, an elevated plasma volume status, and a decreased utilization of loop diuretics (*p* < 0.05 for all).

As an illustrative example, the patient represented in Figure 3A, characterized by an elevated SVI, exhibited a plasma volume status of −9.5% and a cardiac index of 2.44 L/min/m^2^. In contrast, the patient illustrated in Figure 3B, with a low SVI, displayed a plasma volume status of −36.3% and a cardiac index of 1.93 L/min/m^2^.

### 3.3. Prognostic Impact of Baseline SVI

Following the index discharge, the patients were monitored for a median duration of 730 (369, 730) days. During this observation period, two patients experienced cardiac death, and 18 patients faced heart failure readmissions, leading to a total of 18 patients encountering the primary outcome.

Among the baseline variables assessed as potential risk factors for the primary outcome, the SVI, mean right atrial pressure, and pulmonary artery wedge pressure were found to be statistically significant in the univariable analysis (*p* < 0.05 for all; Table 2). In the subsequent multivariable analysis, the SVI was established as an independent risk factor for the primary outcome, with a hazard ratio of 1.09 (95% confidence interval 1.01–1.18, *p* = 0.041), even after adjustment for the mean right atrial pressure and pulmonary artery wedge pressure (Table 2). The SVI remained significant in the multivariable analysis even when age and male sex, both of which may also be potential confounders irrespective of their statistical findings, were included (hazard ratio 1.08, 95% confidence interval 1.01–1.17, *p* = 0.037).

**Figure 4:**
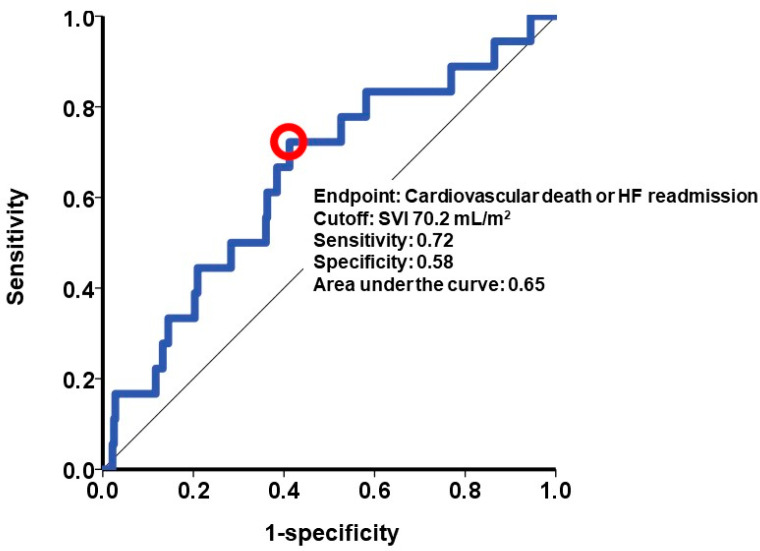
Cutoff of splenic volume index to estimate the primary outcome. The primary outcome was defined as 2-year cardiac death or heart failure readmission. A cutoff of splenic volume index was calculated at 70.2 mL/m^2^ by receiver operating characteristics analysis and shown as a red circle in this curve. SVI, splenic volume index; HF, heart failure.

**Figure 5:**
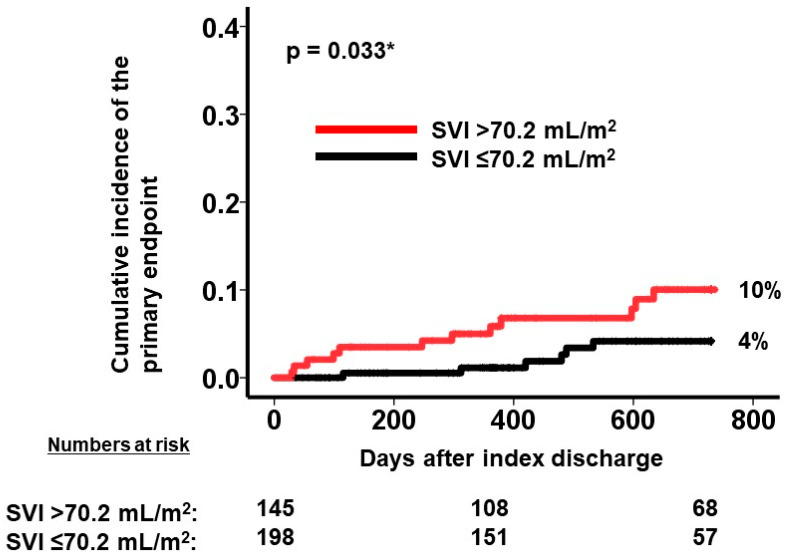
Cumulative incidence of the primary endpoint during 2-year observation period after the index discharge. Cumulative incidence was stratified into two groups by SVI at a cutoff of 70.2 mL/m^2^. A higher SVI was associated with greater cumulative incidence of the primary outcome. SVI, splenic volume index. The primary outcome was defined as cardiac death or heart failure readmission. * *p* < 0.05 by log-rank test.

A calculated SVI cutoff of 70.2 mL/m^2^ (Figure 4) was identified as a predictor for the primary outcome. Out of the patient cohort, 145 individuals possessed SVI values exceeding 70.2 mL/m^2^. The patients with a higher SVI exhibited a significantly greater cumulative incidence of the primary outcome, with rates of 10% compared to 4% in those with a lower SVI (*p* = 0.033; Figure 5).

## 4. Discussion

In this retrospective study, we conducted an assessment of the influence of the baseline SVI levels on the composite endpoint, which encompassed cardiac mortality and readmission due to heart failure during a 2-year observation period following TAVR. The distribution of the baseline SVI exhibited a broad spectrum. Evidently, higher SVI values were linked to advanced systemic volume overload, while lower SVI values were indicative of reduced cardiac output. Importantly, our findings revealed that a higher SVI emerged as an independent predictor for the primary outcome over the 2-year post-TAVR period.

### 4.1. Splenic Size

The concept of the cardio–splenic relationship is a relatively recent development [15]. One of the pivotal roles of the spleen is to function as a buffer for circulating blood [16]. In cases of a hypovolemic state such as cardiogenic shock, the spleen acts to construct and bolster the effective circulating fluid volume [17,18,19,20,21], while in congestive heart failure, it enlarges and strives to conserve fluid volume [22].

The splenic size has emerged as a valuable metric for assessing the splenic function [24]. However, it is important to note that the splenic size can exhibit variability based on factors such as age, gender, race, and body composition [25,26,27]. In line with previous research, we normalized the splenic volume by utilizing the body surface area as a scaling factor. Our study featured elderly Japanese patients, and it is worth acknowledging that the generalizability of our findings, especially with regard to the absolute values and the determination of the SVI cutoff points, to other patient populations remains uncertain. In this study, the SVI values displayed a wide range, akin to previous cohorts involving heart failure, likely attributable to the diversity of hemodynamic profiles [21,28] For example, in a recent study involving a stable heart failure cohort, the median SVI was 68.9 mL/m^2^, which was almost similar to our cohort [21].

### 4.2. Cardio–Splenic Relationship

To explore the connection between the SVI and clinical parameters in our cohort of TAVR candidates, we divided them into tertiles. In a prior study involving a stable heart failure cohort, a lower cardiac output, heightened sympathetic nerve activity, and hypoxia were found to be correlated with a decreased SVI [21]. Notably, the SVI exhibited an increase following enhancements in the cardiac output and the stabilization of sympathetic nerve activity through the implementation of durable left ventricular assist devices [29]. Similarly, in our cohort, a reduction in the cardiac output was linked to a lower SVI. This observation aligns with our patient population, given that many individuals experienced reduced cardiac output due to a stenotic aortic valve, prompting the spleen to contract to augment the effective circulating blood volume.

In contrast, an alternative cohort, characterized by acute decompensated heart failure, demonstrated an association between a higher SVI and systemic congestion [22,30]. Remarkably, we consistently observed in our cohort that systemic congestion, assessed with the plasma volume status, was associated with an elevated SVI. Of note, in the previous stable heart failure cohort, there was no significant link between systemic congestion and the SVI [21]. The correlation between systemic congestion and the SVI may only manifest when the patient cohort exhibits systemic congestion, like our cohort.

### 4.3. Prognostic Impact of Higher SVI

The prognostic impact of the SVI in previous literature has yielded conflicting results, likely owing to the diverse profiles of the cohorts being studied. In a prior investigation involving patients with stable heart failure, a lower SVI was linked to a diminished cardiac output and a heightened risk of cardiac death and heart failure recurrence [21]. It appears that systemic congestion was reasonably well managed within the cohort under study.

In contrast, a different study, which included patients with decompensated heart failure, found that a higher SVI was associated with systemic congestion and subsequently a higher rate of heart failure recurrence [28]. These differing findings further underscore the complexity of the relationship between the SVI and clinical outcomes, which can be influenced by the specific patient population and clinical context under investigation [28].

In our study, we observed that a higher baseline SVI, which was indicative of systemic congestion, was associated with a greater risk of cardiac death or heart failure recurrence following TAVR. This suggests that a degree of systemic congestion may persist even after the TAVR procedure, potentially contributing to the exacerbation of heart failure in affected patients. These findings emphasize the importance of monitoring and managing congestion in TAVR candidates, particularly those with higher SVI values, to improve clinical outcomes [11,12,13,14].

Conversely, the baseline lower cardiac output resulting from a stenotic aortic valve can be effectively ameliorated by TAVR. This might explain why a lower baseline SVI was not associated with worsening clinical outcomes in our study. TAVR can lead to improvements in the cardiac output and alleviate the hemodynamic burden caused by aortic stenosis, mitigating the potential adverse effects of a lower SVI in this context.

### 4.4. Clinical Implication of SVI Measurement

In light of the robust evidence supporting the prognostic advantages of TAVR over medical therapy alone for patients with severe aortic stenosis [1], our findings do not necessarily preclude individuals with a high SVI from undergoing TAVR. However, our study allows us to engage in risk stratification and communicate the potential risks associated with TAVR, especially for patients with a high SVI, during the shared decision-making process with patients and their families prior to the procedure. Importantly, since computed tomography is routinely performed to assess anatomical suitability before TAVR, there is no need for additional testing to calculate the SVI.

The management and clinical implications of an elevated SVI represent the next area of concern. One notable advantage of SVI measurement is that it serves as an indicator of “significant congestion.” Patients’ homeostatic systems detect this significant congestion, prompting the spleen to attempt to store excess fluid volume. This may explain why the other congestion-related parameters were not significantly associated with the clinical outcomes in our study (see our above examples). Given that the patients with a high SVI had a lower prevalence of diuretic use, it raises the possibility that more aggressive diuretic therapy to alleviate relative congestion might lead to improved clinical outcomes after TAVR. The prognostic impact of SVI-guided management and the trajectory of the SVI after TAVR remains the future research direction. The implication of the SVI in a variety of other etiologies is another concern.

## 5. Limitations

This study included a moderately-sized cohort, and we employed non-parametric statistics to analyze the continuous variables, presenting them as medians and interquartile ranges rather than means and standard deviations. While we attempted to account for potential confounders in our analysis to assess the prognostic impact of the SVI, it is plausible that there may be additional unexplored confounders with significant prognostic relevance that were not considered in this study. For example, cardiac amyloidosis is a recently introduced etiology that progresses aortic valve degeneration, impairs cardiac diastolic function, and has a negative prognostic impact even after TAVR. The screening for the presence of cardiac amyloidosis may not be mandatory, and undiagnosed cardiac amyloidosis may have been a potential confounder [31].

Our observation period spanned two years, and the impact of the baseline SVI on longer-term outcomes remains uncertain. However, given the advanced age of TAVR candidates, such as in our cohort, the longer-term prognosis after TAVR may not hold substantial clinical significance.

We did not employ shear-wave elastography, an alternative method for assessing the splenic function by measuring splenic stiffness. In previous research, splenic stiffness was found to correlate with right atrial pressure, and high splenic stiffness was associated with future occurrences of cardiac death or heart failure recurrence in heart failure cohorts [32].

It is noteworthy that we only measured SVI once before TAVR, and the prognostic impact of its longitudinal trend was not evaluated. The size of the spleen can undergo significant changes based on the dynamic course of the hemodynamics, and the impact of aggressive congestion management guided by SVI values remains uncertain. Additionally, the splenic size can vary based on factors such as race, gender, body size, and underlying medical conditions, raising questions about the generalizability of our findings to other patient populations.

## 6. Conclusions

Our study found that a higher baseline SVI, which serves as an indicator of significant systemic congestion, was associated with an increased risk of cardiac death or heart failure readmission following TAVR. The clinical implications and potential utility of SVI-guided management in TAVR candidates warrant further evaluation and investigation to assess its potential benefits in optimizing patient outcomes.

## Figures and Tables

**Table 1:** Baseline characteristics.

	Total (*N* = 343)	First Tertile (*N* = 114)	Second Tertile (*N* = 114)	Third Tertile (*N* = 115)	*p* Value
Demographics					
Age, years	86 (83, 88)	86 (83, 89)	86 (83, 89)	85 (82, 88)	0.35
Male sex	98 (26.8%)	24 (21.1%)	33 (28.9%)	41 (35.7%)	0.050
Body mass index, kg/m^2^	21.7 (19.2, 24.3)	20.9 (18.6, 23.3)	21.8 (19.4, 24.2)	22.3 (19.5, 24.6)	0.049 *
Vital signs					
Systolic blood pressure, mmHg	114 (103, 125)	117 (104, 127)	112 (102, 125)	111 (102, 123)	0.23
Diastolic blood pressure, mmHg	60 (51, 68)	61 (50, 67)	59 (50, 68)	60 (53, 71)	0.69
Pulse rate, bpm	71 (63, 78)	72 (63, 81)	70 (61, 76)	70 (63, 79)	0.20
Comorbidity					
Hypertension	251 (73.2%)	77 (67.5%)	89 (78.1%)	85 (73.9%)	0.19
Diabetes mellitus	61 (17.8%)	20 (17.5%)	17 (14.9%)	24 (20.9%)	0.52
Dyslipidemia	164 (47.8%)	56 (49.1%)	52 (45.6%)	56 (48.7%)	0.86
Atrial fibrillation	56 (16.3%)	15 (13.2%)	19 (16.7%)	22 (19.1%)	0.47
History of cardiac surgery	25 (7.3%)	9 (7.9%)	6 (5.3%)	10 (8.7%)	0.60
History of stroke	45 (13.1%)	11 (9.6%)	20 (17.5%)	14 (12.2%)	0.19
Coronary artery disease	87 (25.4%)	28 (24.6%)	23 (20.2%)	36 (31.3%)	0.16
Laboratory data					
Hemoglobin, g/dL	11.1 (10.0, 12.3)	11.0 (9.8, 12.1)	11.4 (10.2, 12.3)	11.3 (10.0, 12.3)	0.49
Serum albumin, g/dL	3.8 (3.5, 4.0)	3.7 (3.4, 3.9)	3.8 (3.6, 4.1)	3.9 (3.5, 4.0)	0.077
Serum sodium, mEq/L	140 (138, 142)	141 (138, 142)	141 (138, 142)	140 (139, 142)	0.82
Serum potassium, mEq/L	4.4 (4.1, 4.6)	4.3 (4.0, 4.6)	4.5 (4.1, 4.7)	4.3 (4.1, 4.5)	0.33
eGFR, mL/min/1.73 m^2^	49.0 (36.3, 60.1)	47.8 (37.0, 58.4)	49.1 (35.2, 60.3)	50.3 (36.8, 61.9)	0.75
Plasma BNP, pg/mL	221 (119, 483)	195 (123, 497)	282 (141, 507)	186 (102, 474)	0.65
Echocardiography					
LVDd, mm	46 (42, 51)	46 (40, 50)	45 (41, 50)	47 (43, 51)	0.88
LVEF, %	65 (54, 70)	65 (54, 69)	66 (54, 72)	63 (53, 70)	0.24
Moderate or greater MR	10 (2.9%)	1 (0.9%)	4 (3.4%)	5 (4.3%)	0.28
Moderate or greater TR	4 (1.2%)	0 (0.0%)	3 (2.6%)	1 (0.9%)	0.14
Peak velocity at aortic valve, m/s	4.4 (4.0, 4.8)	4.4 (4.0, 4.8)	4.3 (4.0, 4.8)	4.5 (4.1, 4.9)	0.056
Hemodynamics					
Mean right atrial pressure, mmHg	5 (3, 7)	5 (3, 7)	5 (4, 8)	6 (4, 9)	0.058
PAWP, mmHg	12 (9, 16)	12 (8, 16)	12 (9, 15)	12 (9, 16)	0.90
Cardiac index, L/min/m^2^	2.66 (2.29, 2.99)	2.59 (2.27, 2.86)	2.65 (2.25, 3.01)	2.77 (2.40, 3.05)	0.048 *
Plasma volume status, %	7.0 (−5.5, 18.6)	4.5 (−7.8, 15.6)	5.4 (−6.1, 15.4)	11.9 (−0.2, 22.3)	0.007 *
Medications					
Beta-blocker	112 (32.7%)	33 (28.9%)	44 (38.6%)	35 (30.4%)	0.25
Renin–angiotensin system inhibitor	212 (61.8%)	74 (64.9%)	77 (67.5%)	61 (53.0%)	0.055
Mineralocorticoid receptor antagonist	97 (28.3%)	33 (28.9%)	28 (24.6%)	36 (31.3%)	0.52
Loop diuretics	187 (54.5%)	74 (64.9%)	51 (44.7%)	62 (53.9%)	0.009 *
SVI, mg/m^2^	65.5 (48.9, 86.9)	43.8 (35.0, 48.9)	65.5 (60.9, 70.5)	96.0 (86.9, 116)	<0.001 *

SVI, splenic volume index; eGFR, estimated glomerular filtration rate; BNP, B-type natriuretic peptide; LVDd, left ventricular end-diastolic diameter; LVEF, left ventricular ejection fraction; MR, mitral regurgitation; TR, tricuspid regurgitation; PAWP, pulmonary artery wedge pressure. Patient cohort was divided into tertiles according to the baseline value of SVI into low, medium, and high. Continuous variables were stated as median (25% interquartile, 75% interquartile) and compared between the three groups using Kruskal–Wallis test and post hoc Dunn’s test. Categorical variables were stated as numbers (percentage) and compared between the three groups using chi-square test. * *p* < 0.05.

**Table 2:** Impact of SVI on the primary outcome.

	Univariable Analysis		Multivariable Analysis	
	Hazard Ratio (95% CI)	*p* Value	Hazard Ratio (95% CI)	*p* Value
SVI, ×10 mL/m^2^	1.09 (1.01–1.19)	0.032 *	1.09 (1.01–1.18)	0.041 *
Age, years	1.06 (0.96–1.16)	0.27		
Atrial fibrillation	1.73 (0.57–5.27)	0.33		
Plasma B–type natriuretic peptide, pg/mL	1.00 (1.00–1.01)	0.99		
Left ventricular ejection fraction, %	1.01 (0.97–1.04)	0.81		
Mean right atrial pressure, mmHg	1.22 (1.04–1.42)	0.015 *	1.11 (0.88–1.38)	0.38
Pulmonary artery wedge pressure, mmHg	1.10 (1.03–1.18)	0.007 *	1.06 (0.97–1.17)	0.20
Cardiac index, L/min/m^2^	0.47 (0.16–1.38)	0.17		
Plasma volume status, %	0.99 (0.96–1.01)	0.25		
Loop diuretics	2.97 (0.98–9.02)	0.055		

SVI, splenic volume index; CI, confidence interval. Potential prognostic parameters were included in the univariable analysis, including SVI. Variables with *p* < 0.05 in the univariable analysis were included in the multivariable analysis. * *p* < 0.05 by Cox’s proportional hazard ratio regression analysis.

## Data Availability

Data are available upon reasonable request by the corresponding author.

## Appendix A. How to Calculate Estimated Plasma Volume Status

Data pertaining to body weight (BW), body height, and hematocrit (Ht) measurements obtained prior to TAVR were systematically acquired from all study participants. The estimation of plasma volume (PV) was executed employing the Hakim formula, which is articulated as follows:

PV (liters) = [1 − Ht (%)] × [a × (b × (lean body mass))].

Here, “a” stands at 1530 for males and 864 for females, while “b” is 41.0 for males and 47.2 for females. The calculation of lean body mass was ascertained using the subsequent equations:

For males: 0.33 × BW (kilograms) + 0.34 × [body height (centimeters)] − 29.5

For females: 0.30 × BW (kilograms) + 0.42 × [body height (centimeters)] − 43.3

The determination of the PV status was predicated on the ensuing formula:

PV status (%) = [((estimated PV) − (ideal PV)) / (ideal PV)] × 100

The ideal PV (liters) was calculated as “a” times BW (kilograms), with “a” being 39 for males and 40 for females.
